# The Influence of Diet on the Composition and Function of Gut Microbiota in Four Snake Species

**DOI:** 10.1002/ece3.72204

**Published:** 2025-09-25

**Authors:** Huina Song, Xiufeng Li, Jingxue Luo, Ji Wang, Fei Wu, Jiuyan Jiang, Ji Chen, Yuqi Cheng, Yujia Yong, Mingwen Duan, Guangxiang Zhu

**Affiliations:** ^1^ College of Life Science Sichuan Agricultural University Ya'an China; ^2^ Luzhou College of Medical Devices Luzhou China; ^3^ Chengdu Zoo Chengdu China; ^4^ School of Ecology and Environment, Tibet University Lhasa Tibet China

**Keywords:** diet, gut microbiota, metagenome, snakes

## Abstract

Diet plays an important role in shaping the intestinal flora, especially as demonstrated in non‐mammalian studies. However, the precise associations underlying the interaction between the snake gut microbiota and diet remain poorly understood. Our findings indicate that the gut microbiomes of four snake species exhibit distinct characteristics influenced by their dietary preferences. Significant variations in gut microbial composition were observed among snakes with different diets. Similarities were noted between the gut microbiomes of Jerdon's pitviper ( *Protobothrops jerdonii* : PJ) and Black‐browed ratsnake (*Elaphe taeniura*: ET), which share similar food preferences. Chinese slug‐eating snake ( *Pareas chinensis* : PC), which primarily feeds on snails and slugs, displayed the highest gut microbiota diversity, suggesting a higher level of functional specificity associated with its specialized diet. Chiwen keelback (*Rhabdophis chiwen*: RC), which consumes fireflies and earthworms, exhibited a significantly higher abundance of antimicrobial resistance genes (ARGs) compared to other snake groups. Opportunistic pathogens such as *Plesiomonas*, *Aeromonas*, and *Salmonella* were relatively abundant in RC, ET, and PJ. The results of this study provide comprehensive data to differentiate the gut microbiota composition structures among four snake species with distinct dietary preferences, explore their potential functions, and identify possible correlations between gut microbial composition and diet. Furthermore, it provides a foundation for the analysis of the influence of genetic and environmental factors on the evolution of gut microbial communities.

## Graphical abstract

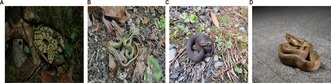

## 1 Introduction

The gut microbiota is the most important “microbial organ” in the animal organism and plays an important role in the digestion and absorption of nutrients in the host (Ahern et al. 2014; Hooper et al. 2012; Malmuthuge et al. 2015; O'Mahony et al. 2015; Sylvia et al. 2017). Thus, the gut microbiota may be an important determinant of individual growth, development, immunity, and survival (Hu et al. 2018; Rosshart et al. 2017; Wang et al. 2024; Yao et al. 2022).

Phylogenetic relationships and diet exert significant influences on gut microbiome composition (Wu et al. 2022). Diet acts as a key modifiable exogenous factor, driving substantial short‐term fluctuations in microbiome diversity and structure (Leeming et al. 2019). However, long‐term dietary patterns demonstrate a more profound impact than short‐term dietary intake (Hird et al. 2015). Specifically, meat‐centric diets high in protein and fat are strongly associated with distinct alterations in the gut microbiome (Johnson et al. 2019). Dietary modifications induce subtle yet significant shifts in microbial communities even in non‐mammalian hosts, exemplified by the insectivorous house swallow ( *Hirundo rustica* ) (Schmiedová et al. 2022). Furthermore, dietary influences on the gut microbiota are evident in species with specialized feeding ecologies, including lizards and bat species (Gong et al. 2021; Jiang et al. 2017). Thus, diet is widely recognized as a key driver of microbiome change. Simultaneously, the gut microbiome is recognized as a critical reservoir for antibiotic resistance genes (ARGs). ARGs shed in animal feces disseminate through environmental matrices like soil and water, posing ecological risks (Joyce et al. 2019). Selective pressures from animal diets or direct antibiotic exposure amplify ARG enrichment, potentially facilitating transmission to humans via the food chain and presenting public health threats (Da Silva et al. 2021; Kumar et al. 2020). In addition, long‐term dietary habits act on the abundance of ARGs by altering the gut microbiota (Liang et al. 2025), and the intake of high‐fiber and plant‐based foods inhibits the transmission of ARGs (Liu et al. 2019; Theophilus and Taft 2023).

Snakes exhibit diverse diets, have a wide range, are adaptable to diverse habitats, and are currently represented by more than 4000 species (Grundler and Rabosky 2021; Zhao 2006). Snakes compensate energetically by swallowing their prey whole at once and digesting it rapidly (Glaudas et al. 2019). The gastrointestinal physiology of snakes is fascinating due to their remarkable ability to withstand extended periods of fasting, resulting in a reduction in the mass of gastrointestinal organs and the maintenance of metabolic costs at a lower rate. Interestingly, snakes can quickly reestablish their capacity to digest and absorb food immediately after consuming prey, even in the absence of regular feeding (Bury 2022; Enok et al. 2013; Holmberg et al. 2002; Secor 2008; Secor and Diamond 2000; Wang and Rindom 2021). The remarkable physiological adaptations observed in snakes, influenced by evolution and environmental factors, are consistent with the Krogh principle of comparative physiology. Consequently, snakes possess a high level of efficiency in absorbing nutrients (Holmberg et al. 2002). Previous studies on *Python bivittatus* revealed that the gut microbiomes of these snakes change digestion, showing an observable increase in the abundance and diversity of Firmicutes (Costello et al. 2010). The enzymes produced by these phyla groups play a crucial role in energy metabolism and can break down a diverse array of large molecules found in food, thereby offering a valuable nutrient source to the host (Fernandes et al. 2014; Zhao et al. 2018). However, there is still a serious lack of research on the relationship between snake diet and microbiota and ARGs.

**FIGURE 1:**
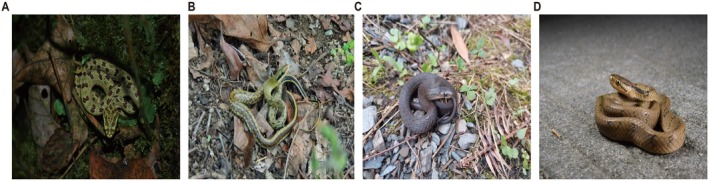
Color photographs of four snake species: *Protobothrops jerdonii* (A), *Elaphe taeniura* (B), *Rhabdophis chiwen* (C), *Pareas chinensis* (D). Image credits: Photos A and B were provided by Rui Zhou; Photo C was provided by Mingwen Duan; Photo D was provided by Junjie Huang and Ke Jiang.

This study investigated the gut microbial diversity of the Chinese slug‐eating snake ( *Pareas chinensis* ), Black‐browed ratsnake (*Elaphe taeniura*), Chiwen keelback (*Rhabdophis chiwen*), and Jerdon's pitviper ( *Protobothrops jerdonii* ) with three different dietary preferences using metagenomics (Figure 1). *P. chinensis* (Family: Pareidae) feeds on snails and slugs (Hoso 2017; Wang et al. 2020). *E. taeniura* (Family: Colubridae) feeds on rodents and birds, has a strong appetite, and consumes a large amount of food, and is commonly seen swallowing 3–4 mice in a row in snake parks (Zhao 2006). *R. chiwen* (Family: Colubridae) feeds on fireflies and earthworms (Fukuda et al. 2022; Piao et al. 2020; Yoshida et al. 2020). *P. jerdonii* (Family: Viperidae) feeds on birds, mice, and small insectivorous mammals (Zhao 2006). Furthermore, the vertebrate‐feeding *E. taeniura* and the invertebrate‐feeding *R. chiwen* belong to the same family but have very different diets. *E. taeniura* and *P. jerdonii* belong to different families, but both feed mainly on vertebrates. This allows us to maximize the use of natural variation and effectively resolve the key drivers of diet on gut flora structure and function. We predict that the results of the study will contribute to a better understanding of the intestinal digestive patterns, food habits, and health status of wild snakes, reveal the associations of adaptation of their gut microbiota to the host environment by providing basic data, and increase the understanding of the intestinal microbiota of wild snakes.

## 2 Materials and Methods

### 2.1 Sample Collection and DNA Extraction

During the summer of 2022, specimens of *Pareas chinensis* (PC), *Rhabdophis chiwen* (RC), and *Protobothrops jerdonii* (PJ) were collected in Sichuan Province, China, while *Elaphe taeniura* (ET) specimens were collected in Shandong Province, China, based on morphological identification. Due to challenges in procuring wild individuals, three biological replicates per species were included in this study, yielding a total of 12 samples. Each collected snake was placed in a separate sterile plastic bottle and transported to the Zoology Laboratory of Sichuan Agricultural University. Intestinal contents provide a more accurate representation of in vivo gut microbial communities than fecal samples (Sun et al. 2024). In this study, we fasted the snakes and ensured that their prey had been completely digested by hand. We then euthanized the snakes and extracted their intestinal contents separately from the large intestine. This study was reviewed and approved by the Institutional Animal Care and Use Committee of Sichuan Agricultural University (Approval No. 20210071), and no endangered or protected species were used. The gut contents were promptly transferred to sterile 2 mL collection tubes and immediately frozen in liquid nitrogen for rapid freezing and then stored at −80°C.

### 2.2 Library Construction, Metagenome Sequencing, and Quality Control

DNA was extracted from approximately 1 g of gut content sample using a Magnetic Soil and Stool DNA Kit (Li et al. 2022), tested for purity, integrity, and concentration using agarose gel electrophoresis (AGE) and Qubit 2.0 (Invitrogen, USA), and then stored frozen at −80°C. High–quality DNA samples were randomly fragmented into approximately 350 bp fragments using an ultrasonic crusher (Covaris, UK). Subsequently, end repair was performed, and adapters containing barcode regions were ligated to both ends of the fragmented DNA to distinguish different samples. Finally, the intact ligation products were isolated, and PCR amplification was carried out using primers to increase the sequencing library volume and enrich the products with successfully ligated adapters. Following library construction, preliminary quantification was conducted using Qubit 2.0 (Invitrogen, USA). It is important to note that negative controls were not included during the DNA extraction and library preparation steps in this study. The library was then diluted to 2 ng/μL, and the insert size of the library was assessed using an Agilent 2100 (Agilent, USA). Once the insert size met expectations, qPCR was utilized to precisely quantify the effective concentration of the library (> 3 nM) to ensure its quality. After passing the library check, pooling was performed based on the effective concentration and the desired downstream data volume, followed by sequencing using Illumina PE150. Following the sequencing process, a proportion of low‐quality data was identified in the raw sequencing output. Metagenomic sequencing library construction and Illumina sequencing were performed by Novogene Co. Ltd. (Beijing, China). To guarantee the precision and dependability of subsequent analyses, quality control procedures were implemented for the raw sequencing data. These included the removal of reads containing more than a certain percentage of low‐quality bases (quality value <=38) (the default value is 40 bp), reads containing N bases of 10 bp, and reads with an overlap of more than 15 bp with the adapter. Furthermore, to gain additional insight, snake reference genomes (assembly HabAm_1.0, GCA_003402635.1; assembly CU_Pguttatus_1, GCF_029531705.1; assembly DSBC_Tbai_1.0, GCA_003457575.1; assembly P. Mucros_1.0, GCF_001527695) were compared. The assembly Cadam_11369 (GCA_018446365.1) was compared to remove reads that may have originated from the host (Bowtie 2, parameter settings: ‐end‐to‐end, ‐sensitive, ‐X 400, ‐I 200) (Karlsson et al. 2013).

### 2.3 Metagenomic Bioinformatics and Statistical Analysis

Clean data was obtained after preprocessing, and clean data was assembled using MEGAHIT (1.0.4) (Scher et al. 2013). ORF (Open Reading Frame) prediction was performed using MetaGeneMark (2.10) (Karlsson et al. 2012). The ORF predictions assembled for each sample were de‐redundant using CD‐HIT, clustered with an identity of 95% and coverage of 90%, and the longest sequences were selected as representative sequences to construct a non‐redundant gene catalog (Fu et al. 2012). The gene abundance of each sample was calculated using the Bowtie2 comparison. To eliminate biases caused by sequencing depth and gene length, we filtered genes with ≤ 2 supporting reads across all samples and applied a TPM‐like algorithm to compute relative gene abundances. Subsequently, samples were normalized by scaling relative abundances to a common threshold, generating a uniformly scaled gene abundance table. Based on the abundance of information on each gene in each sample, basic information statistics, and the number of genes in each sample were analyzed in a Venn diagram analysis was performed. The gene catalog and MicroNR libraries were cross‐referenced to acquire species annotation information for each gene (Unigenes). This information was then integrated with the gene abundance table, resulting in the generation of the species abundance table across various taxonomic levels (Zeller et al. 2014). In a similar vein, functional annotations of unigenes were acquired from the KEGG and CAZymes databases (blastp, eval ≤ 1e‐5) (Buchfink et al. 2015). We assessed alpha diversity (species level) using Kruskal‐Wallis tests, followed by pairwise Wilcoxon rank‐sum tests. For beta diversity, clustering analysis was performed based on Bray‐Curtis distances, and non‐metric multidimensional scaling (NMDS) was used to visualize and compare differences in the gut microbial community structures among snake species. To identify taxonomic biomarkers exhibiting significant abundance differences between groups, we performed Linear Discriminant Analysis Effect Size (LEfSe) analysis at the phylum, genus, and species levels (LDA > 4). We visualized the relative abundances of KEGG and CAZy using bar plots. We then detected significant differences in these functional abundance profiles between groups using LEfSe and MetaStats analyses. Finally, box plots were used to show the differences in the number of antibiotic resistance genes (ARO) between different groups. Circos was employed to display the relative abundance of ARO in different groups. R4.1.2 was primarily employed for analysis and visualization tasks, including species composition histograms, heatmaps, and Circos plots. Additionally, SPSS 27.0 was utilized for certain data tests and plot visualizations.

## 3 Results

### 3.1 Sequencing Data Analysis

**FIGURE 2:**
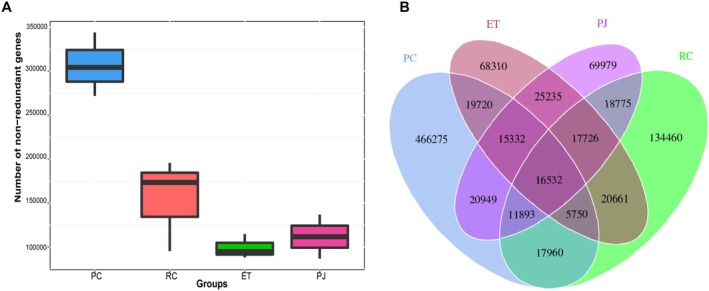
A box plot of the number of genes in the gut microbiota of four snakes; (B) Petal map of the number of genes in the gut microbiota of four snakes. ET, *Elaphe taeniura*; PC, *Pareas chinensis*; PJ, *Protobothrops jerdonii*; RC, *Rhabdophis chiwen*.

Following the extraction of DNA from each gut sample, the samples were subjected to metagenomic sequencing analysis. The results demonstrated that RC yielded the greatest number of sequencing reads, followed by ET, PC, and PJ (Table S1). Following quality control and host genome filtering, pure reads for subsequent analyses were obtained for each sample, with PC obtaining the greatest number of fragments (Table S2). ORF prediction and abundance analyses were conducted on the assembled metagenomes, with the results referenced in Table S3. To better understand the differences in the number of genes between different groups, box plot analysis of the differences in the number of genes between groups revealed that PC had the highest number of unique genes, with a number of 466,275, while the RC, ET, and PJ had 134,460, 68,310, and 69,979, respectively (Figure 2A). However, Venn diagram analysis revealed that the ET and PJ, which have similar diets, have the highest number of overlapping genes at 74,825, which is more than the number of genes unique to each (Figure 2B).

### 3.2 Diversity and Composition of the Gut Microbiota

**FIGURE 3:**
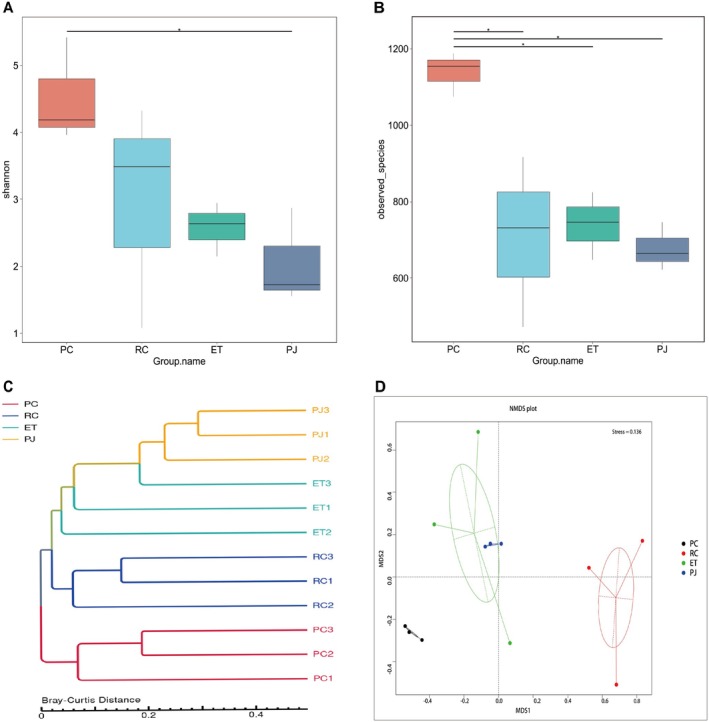
Alpha diversity reflected by Shannon index (A) and Observed_species (B). Cluster tree generated based on Bray–Curtis distance (C). Beta diversity based on NMDS analysis of Bray–Curtis distance (D). Statistical significance was determined using the Wilcoxon test; *p < 0.05. ET, *Elaphe taeniura*; PC, *Pareas chinensis*; PJ, *Protobothrops jerdonii*; RC, *Rhabdophis chiwen*.

The abundance of genes was then combined with species classification to determine abundance at each respective taxonomic level. Sequences were assigned to bacteria, eukaryotes, archaea, and viruses. Bacterial abundance was the highest among the annotated taxonomic species, with percentages ranging from 82.33% to 94.67% across the different samples. Eukaryotes were the second most prevalent group in PC and PJ, while viruses were the second most prevalent group in the gut microbiota of RC and ET (Table S4). No other species were detected in the same batch of extracts or the same batch of build‐ups, leading to the hypothesis that there could be an infection present in the gut of these samples. After obtaining non‐redundant gene annotations by the assembly, the use of the Shannon index and observed species at the genus taxonomic level for the four groups of snakes Analysis of the alpha diversity of the gut microbiota composition of the four groups of snakes revealed that the Shannon and observed species of the PC group were significantly higher than those of the RC, ET, and PJ groups (Wilcox, *p* < 0.05), indicating that the diversity of the PC was the richest and that there were differences in gut microbiota among the four different food characteristics snakes (Figure 3A,B). Cluster tree analysis based on Bray–Curtis distances indicated that samples within the same group exhibited close clustering, except sample ET. This finding suggests that bacterial communities showed minimal variations within each sample group. Moreover, the intestines of the ET and PJ groups displayed greater similarities than those of the other two groups (Figure 3C). NMDS plots showed significant clustering among the different groups and significant overlap between PJ and ET, with smaller distances and tighter clustering among the individuals in the PJ and ET groups (Figure 3D).

**FIGURE 4:**
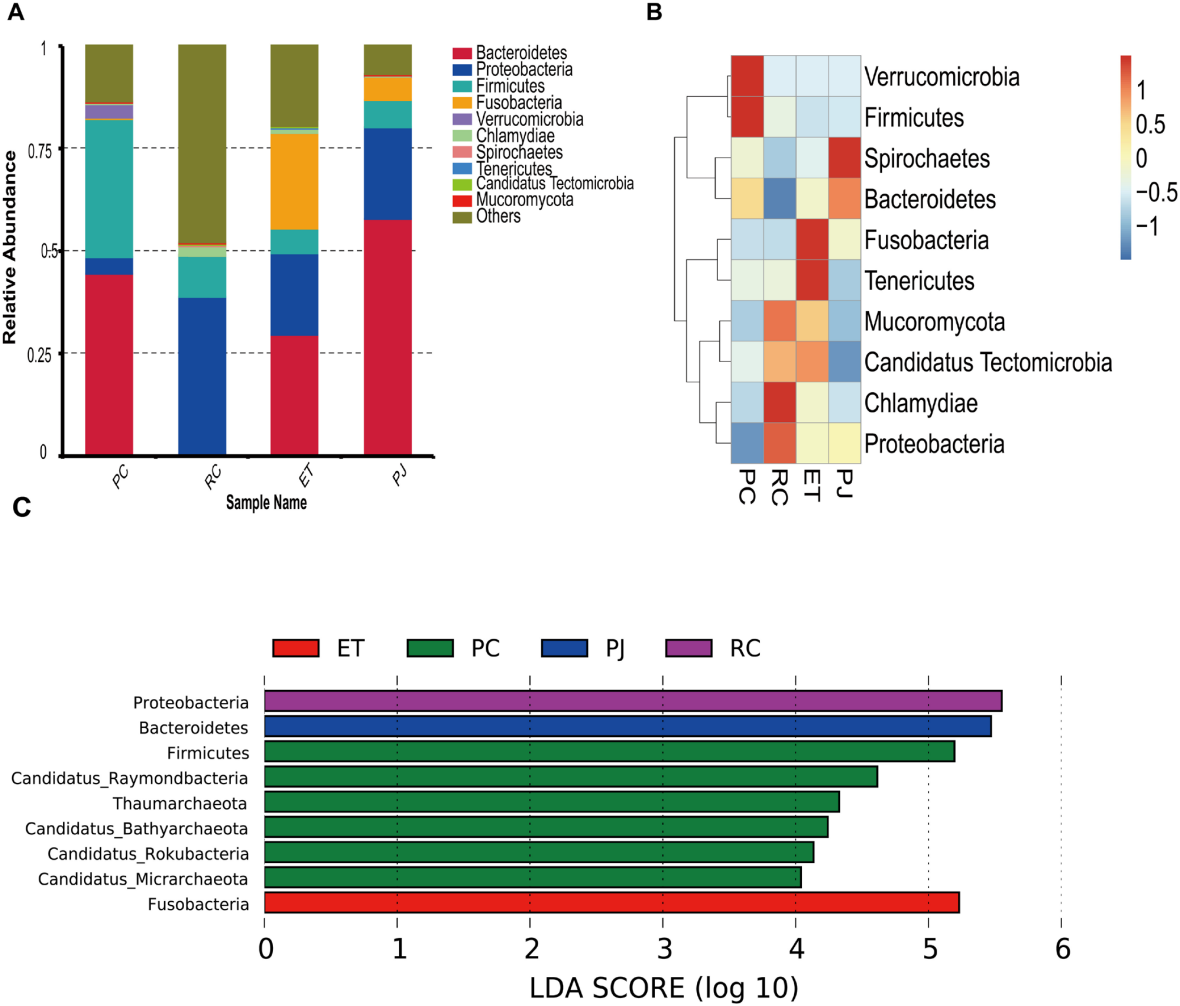
(A) Relative abundance of four snake gut microbiota at the phylum level; (B) Clustered heat map of relative abundance of four snake gut microbiota at the phylum level. (C) Distribution of Linear Discriminant Analysis (LDA) values for species differing in the gut microbiota of four snakes at the phylum (LDA score > 4). ET, *Elaphe taeniura*; PC, *Pareas chinensis*; PJ, *Protobothrops jerdonii*; RC, *Rhabdophis chiwen*.

The dominant phyla in PC were Bacteroidetes (44.30%) and Firmicutes (33.40%); the dominant phyla in RC were Proteobacteria (38.38%) and Firmicutes (10.04%); and the dominant phyla in ET were Bacteroidetes (29.23%), Fusobacteria (23.44%), and Proteobacteria (20.13%) (Figure 4A). The ratios of Firmicutes and Bacteroidetes (F/B) were 0.77, 32.05, 0.21, and 0.12 in PC, RC, ET, and PJ, respectively. The ratio of Firmicutes to Bacteroidetes (F/B) and the relative abundance of Chlamydiae, Mucoromycota, and Candidatus Tectomicrobia were found to be significantly higher in the RC group in comparison to the other groups. Furthermore, the relative abundance of Verrucomicrobia and Bacteroidetes was significantly higher in the PC group than in the other groups. The relative abundance of Fusobacteria, Tenericutes, Mucoromycota, and Candidatus Tectomicrobia was significantly higher in the ET group compared to the other groups. Furthermore, the relative abundance of Spirochaetota and Bacteroidetes was significantly higher in the ET and PJ groups compared to the other groups (Figure 4B). ET, PJ, and RC each have one biomarker; PC has six biomarkers (Figure 4C).

**FIGURE 5:**
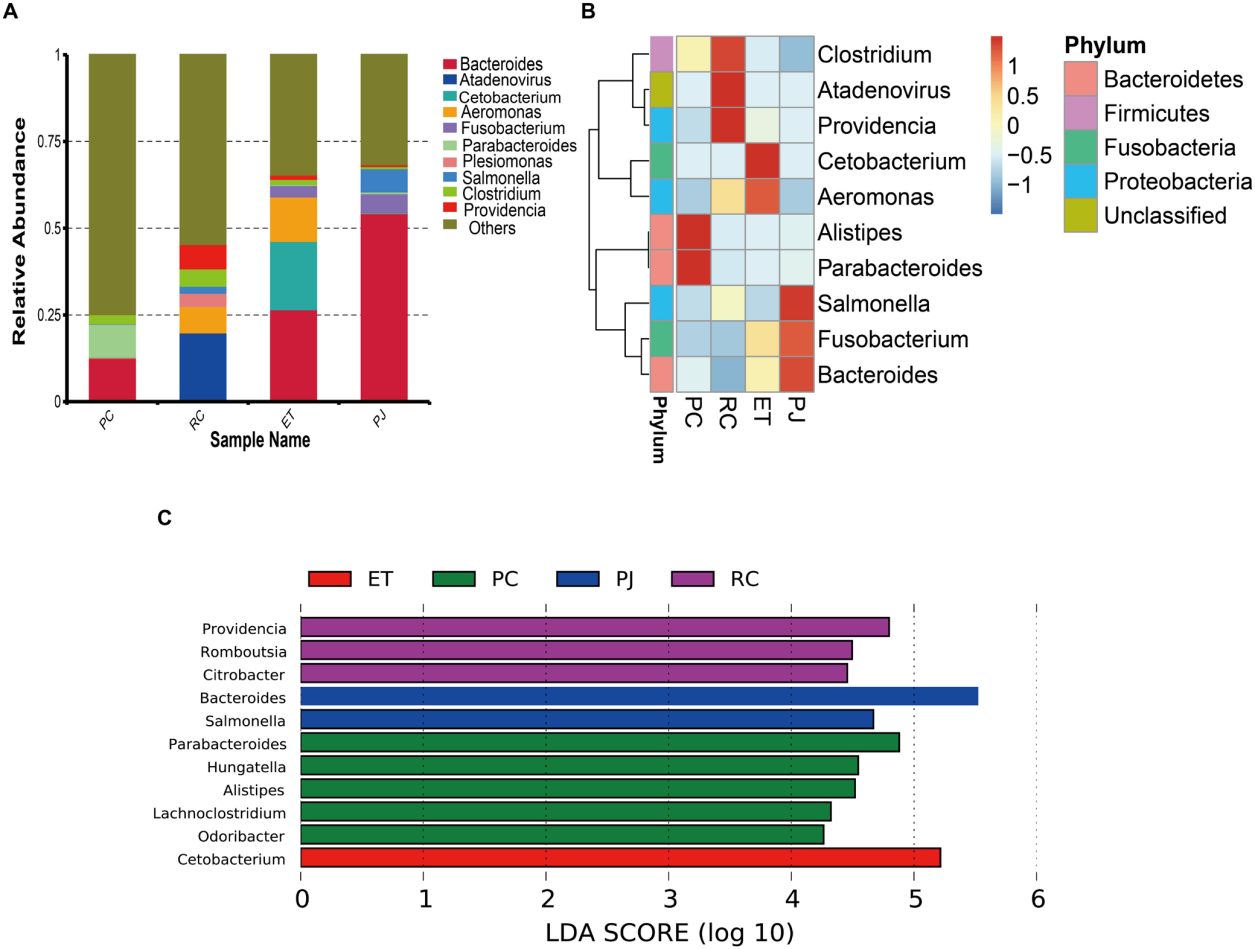
(A) Relative abundance of four snake gut microbiota at the genus level; (B) Clustered heat map of relative abundance of four snake gut microbiota at the genus level; (C) Distribution of Linear Discriminant Analysis (LDA) values for species differing in the gut microbiota of four snakes at the genus (LDA score > 4). ET, *Elaphe taeniura*; PC, *Pareas chinensis*; PJ, *Protobothrops jerdonii*; RC, *Rhabdophis chiwen*.

In this study, intestinal flora with a relative abundance greater than 5% were classified as dominant bacterial genera. The dominant genus composition in the PC group consisted of *Bacteroides* (12.56%) and *Parabacteroides* (9.54%) in that order. In the RC group, the dominant bacterial genera were *Atadenovirus* (19.87%), *Aeromonas* (7.60%), *Providencia* (7.03%), and *Clostridium* (5.02%). Notably, *Atadenovirus* was most abundant in the RC2 samples, suggesting a possible intestinal infection. Upon checking, no contamination was found in the same batch of extractions or libraries. For the ET group, the dominant genera were *Bacteroides* (26.58%), *Cetobacterium* (19.71%), and *Salmonella* (12.80%). In the PJ group, the dominant bacterial genera were *Bacteroides* (54.26%), *Salmonella* (6.69%), and *Fusobacterium* (5.60%) (Figure 5A; Table S5). To better understand genus‐level gut microbial differences, LEfSe analyses were performed, and 11 biomarkers were identified. *Cetobacterium* and *Aeromonas* were relatively abundant in the ET group. Notably, *Cetobacterium* was significantly more abundant than in the other groups. In the PC group, *Alistipes* and *Parabacteroides* were relatively abundant, with *Parabacteroides* being significantly more abundant compared to the other groups. *Bacteroides*, *Fusobacterium*, and *Salmonella* were relatively abundant in the PJ group. Among these, *Bacteroides* and *Salmonella* were significantly more abundant than in the other groups. In the RC group, *Atadenovirus*, *Providencia*, and *Clostridium* were relatively abundant. *Providencia*, in particular, was more abundant than in the other groups (Figure 5B,C).

**FIGURE 6:**
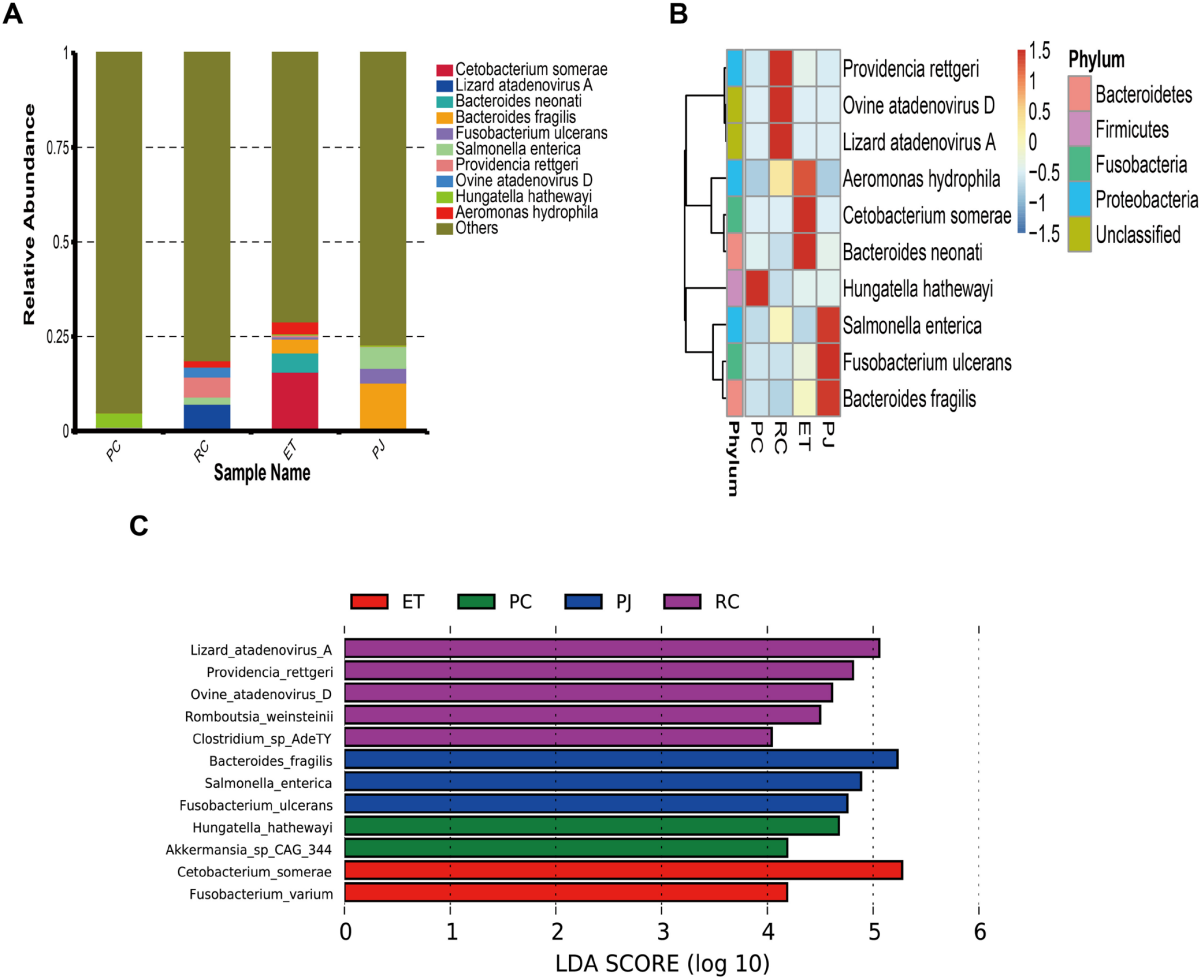
(A) Relative abundance of four snake gut microbiota at the species level; (B) Clustered heat map of relative abundance of four snake gut microbiota at the species level; (C) Distribution of LDA values for species differing in the gut microbiota of four snakes at the species (LDA score > 4). ET, *Elaphe taeniura*; PC, *Pareas chinensis*; PJ, *Protobothrops jerdonii*; RC, *Rhabdophis chiwen*.

At the species level, the classification of snake intestinal bacterial phyla with relative abundance greater than 1% into dominant genera is as follows: the dominant bacteria of PC were in the order of *Hungatella hathewayi* (3.76%); the dominant bacterial species of RC were in the order of *Lizard atadenovirus A* (7.26%), *Providencia rettgeri* (5.26%), *Ovine atadenovirus D* (2.64%), *Salmonella enterica* (1.92%), *Aeromonas hydrophila* (1.63%); the dominant bacterial species of ET were in the order *Cetobacterium somerae* (5.76%), *Lizard atadenovirus A* (5.04%), Bacteroides *neonati* (5.04%), *Bacteroides fragilis* (3.67%), *Aeromonas hydrophila* (3.10%); and the dominant bacterial species of PJ were in the order *Bacteroides fragilis* (12.32%), *Salmonella enterica* (5.75%), *Fusobacterium ulcerans* (3.88%) (Figure 6A). *Providencia rettgeri* , *Ovine atadenovirus D*, *Lizard atadenovirus A* were relatively abundant in RC, *Aeromonas hydrophila* , *Cetobacterium somerae* , *Bacteroides neonati* were relatively abundant in ET, the relative abundance of *Hungatella hathewayi* in PC, and the relative abundance of *Salmonella enterica*, *Fusobacterium ulcerans* , and *Bacteroides fragilis* in PJ (Figure 6B). Species‐level data were analyzed for LEfSe, and biomarkers were 12 (Figure 6C).

### 3.3 Metabolic Potential Functions of Gut Microbiota According to Database

**FIGURE 7:**
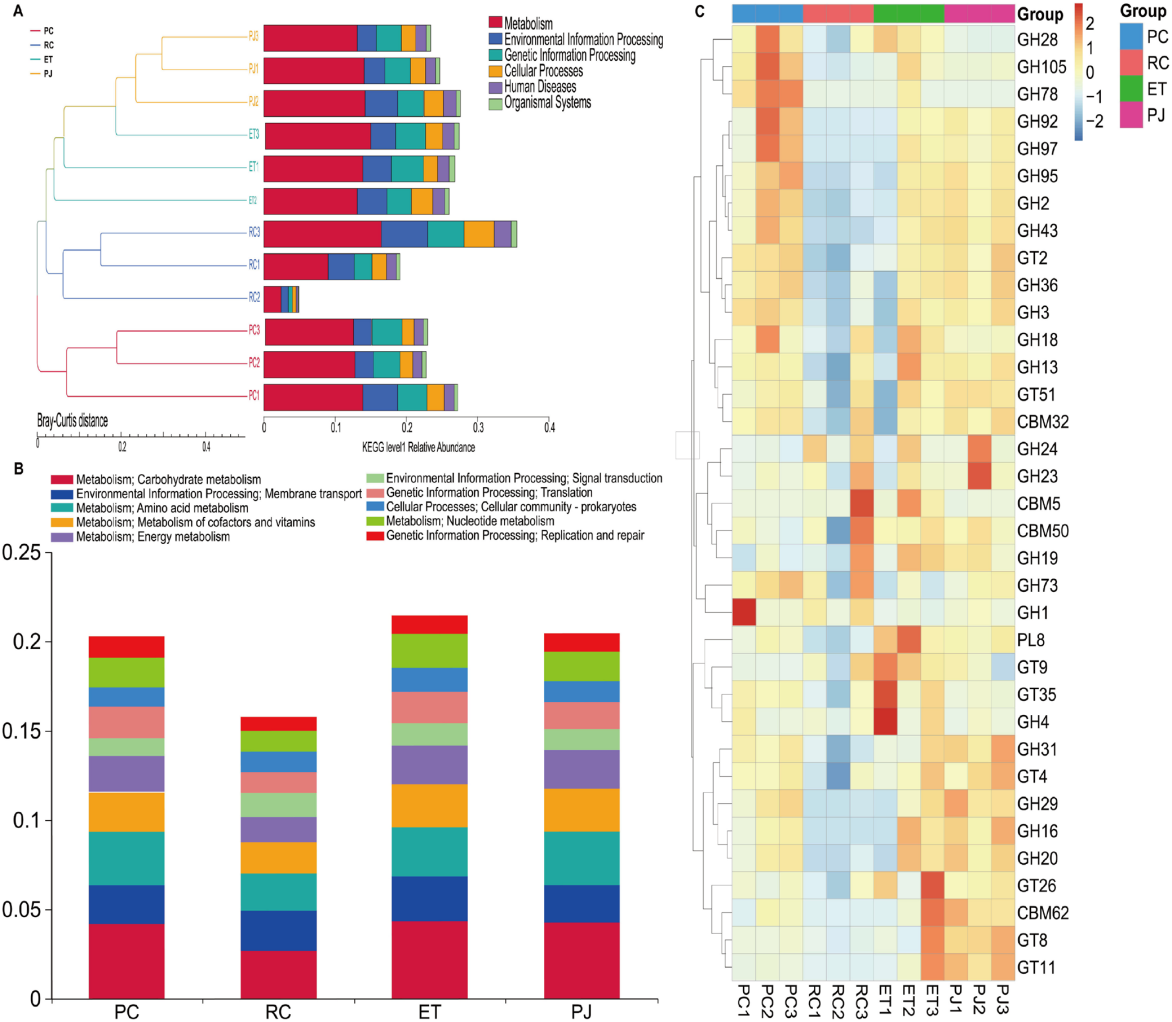
(A) Abundance clustering plot showing the relative functional abundance distribution of KEGG level 1; (B) Bar graph showing the relative functional abundance distribution of KEGG level 2; Heat map showing the relative abundance clustering of Carbohydrate‐Active Enzyme (CAZy) level 2 (C). Horizontal axis: Sample name. Vertical axis: Species information. The values corresponding to the heatmap are Z‐values obtained by normalizing the relative abundance of species in each row. ET, *Elaphe taeniura*; PC, *Pareas chinensis*; PJ, *Protobothrops jerdonii*; RC, *Rhabdophis chiwen*.

Functional annotations of gut microbiota in the KEGG database were examined, and the biological functions associated with these annotations were classified into six distinct groups. In these groups, most of the genes were mapped to metabolism, followed by genes mapped to genetic information processing and environmental information processing (Figure 7A). In KEGG secondary, carbohydrate metabolism, amino acid metabolism, and cofactor and vitamin metabolism were the most abundant pathways (Figure 7B), suggesting that carbohydrates and proteins are more important functional substances in snakes, which are also high‐protein, high‐fat eaters. In CAZy, seven of GH1, GH18, GH28, GH78, and GH97 were significantly higher in PC, CBM5, CBM50, GH19, and GH73 were significantly higher in RC, while 8 and 10 enzymes were higher in each of ET and PJ, of which three were the same and most of the enzymes were similar in abundance in both (Figure 7C), and most of these enzymes were involved in carbohydrate metabolism and amino acid metabolism.

**FIGURE 8:**
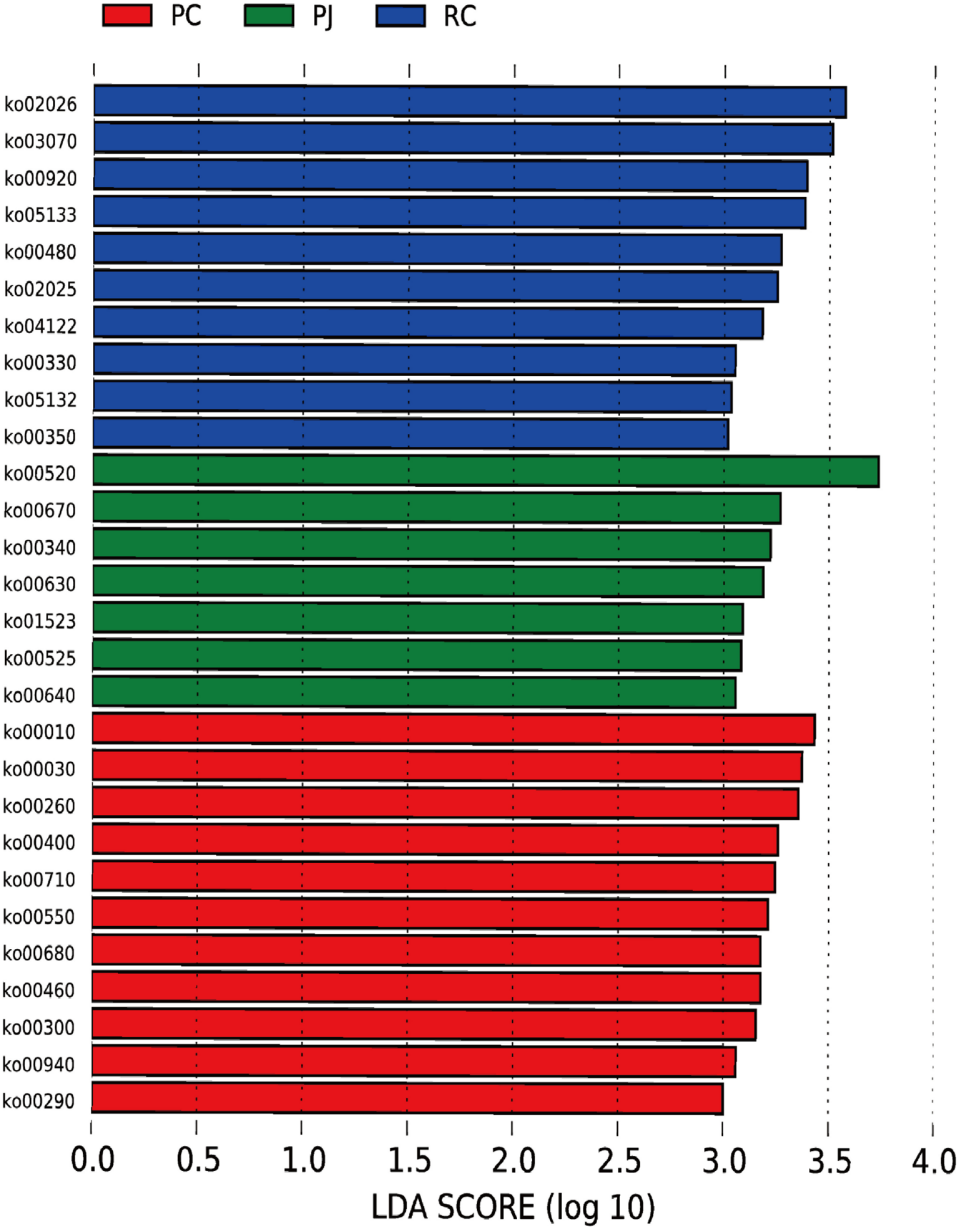
Distribution of LDA scores (> 3) for differential function (KEGG level 3). ET showed no significantly enriched pathways and was not displayed. ET, *Elaphe taeniura*; PC, *Pareas chinensis*; PJ, *Protobothrops jerdonii*; RC, *Rhabdophis chiwen*.

**FIGURE 9:**
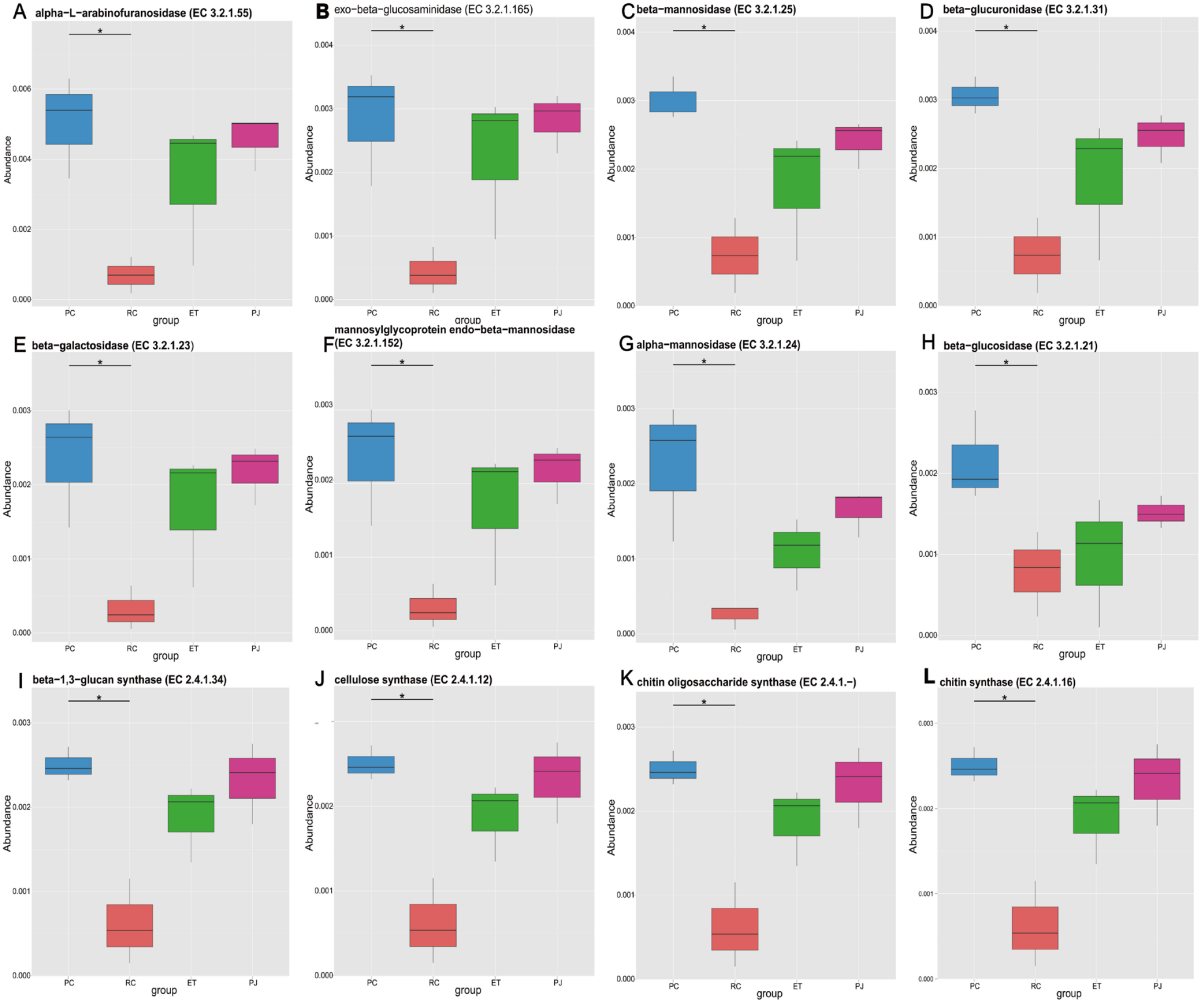
Box plot of CAZy family abundance across significantly different species, categorized by Enzyme Commission (EC) numbers. Statistical significance was determined using Metastats; *p < 0.05. ET, *Elaphe taeniura*; PC, *Pareas chinensis*; PJ, *Protobothrops jerdonii*; RC, *Rhabdophis chiwen*.

To further explore the relationship between food characteristics and gut microbes, LEfSe analysis (LDA score > 3, *p* < 0.05) was performed on KEGG3 levels, and 28 pathways were found to differ between PC, RC, and PJ. As part of our study on food characteristics and gut habits, our primary focus was on examining the carbohydrate metabolism and amino acid metabolism pathways associated with food digestion and absorption. In the carbohydrate metabolic pathway, PC was more abundant in glycolysis/glycolysis (ko00010) and pentose phosphate metabolism (ko00030), and PJ was more abundant in aminosugar and nucleotide sugar metabolism (ko00520), glyoxylate and dicarboxylic acid metabolism (ko00630), and propionic acid metabolism (ko00640). In the amino acid metabolic pathway, PC was more abundant in glycine, serine, and threonine metabolism (ko00260), phenylalanine, lorine, and tryptophan biosynthesis (ko00400), cyanogenic amino acid metabolism (ko00460), lysine biosynthesis (ko00300), and valine, leucine, and isoleucine biosynthesis (ko00290), and RC is more abundant in glutathione metabolism (ko00480), arginine and proline metabolism (ko00330), tyrosine metabolism (ko00350), and PJ is more abundant in histidine metabolism (ko00340) (Figure 8). To deeply investigate the relationship between the metabolic function of the gut microbiota and food characteristics, we analyzed four sets of samples CAZy (ec) by using MetaStats. In CAZy, multiple enzymes differed between the PC and RC groups, with two (EC 2.4.1.–, EC 2.4.1.16) found in the top 12 enzymes contributing to chitinase synthesis, one contributing to cellulase synthesis (EC 2.4.1.12), and a variety of enzymes favoring the degradation of large molecules of starch and sugar into small molecules (Figure 9).

### 3.4 Relationship Between Gut Microbiota and Antibiotic Resistance Genes

**FIGURE 10:**
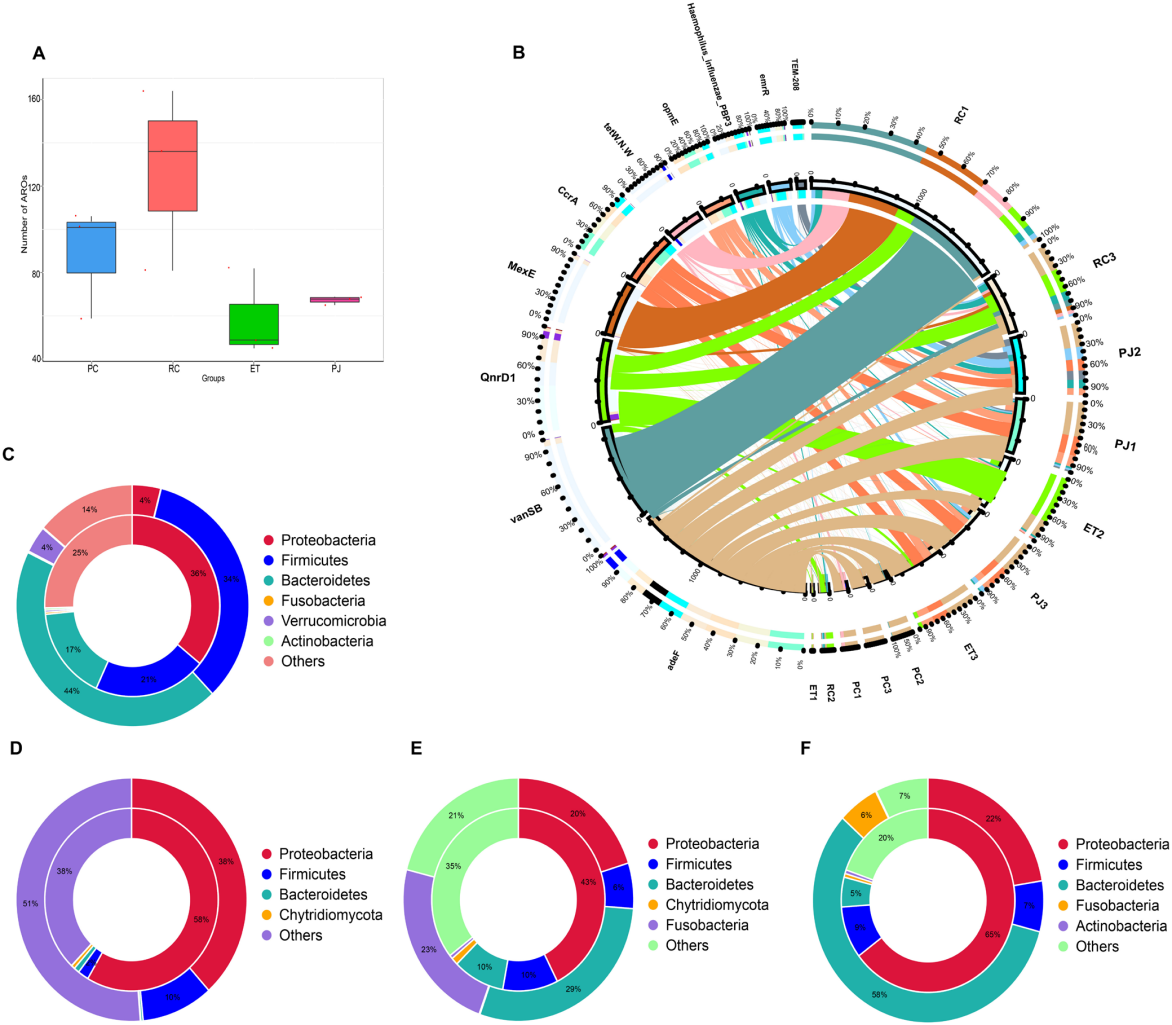
Inter–group ARO number box diagram (A); Top 10 ARO abundance circles in samples (B); Two‐circle of PC (C), RC (D), ET (E), and PJ (F) resistance genes about species affiliation. Note B: The circle diagram is divided into two parts; the right side is sample information, and the left side is ARO information. Different colors in the inner circle indicate different samples and AROs, with the sum of the relative abundance in individual samples of an ARO on the left side and the sum of the relative abundance of each ARO in a given sample on the right side, and the relative percentage of individual samples in a given ARO on the left side of the outer circle and a given sample on the right side of the outer circle. Notes C, D, E, F: In the circle diagram for species attribution analysis of resistance genes, the inner circle shows the species distribution of AROs, and the outer circle shows the species distribution of all sample genes in the group. ET, *Elaphe taeniura*; PC, *Pareas chinensis*; PJ, *Protobothrops jerdonii*; RC, *Rhabdophis chiwen*.

This section outlines a study conducted to examine the occurrence of ARGs in the gut microbiota of four distinct groups of snakes based on their food characteristics. The analysis involved the identification and evaluation of individual genes within the metagenomic data for potential antibiotic resistance factors using the Comprehensive Antibiotic Resistance Database (CARD). According to the CARD annotation results, the snake population with the highest and most abundant antibiotic resistance ontologies (AROs) was observed in the RC population when compared to the other three snake populations (Figure 10A, Table S6), and it is suggested that there may be a correlation between higher levels of ARGs in the soil and the properties of the food consumed by earthworms. Furthermore, analysis of the species network map revealed that the top 10 resistance genes were predominantly related to tetracycline and fluoroquinolone antibiotics, and were found in higher quantities within PC. Nearly 86% of the AROs were highly enriched in Bacteroidetes (44%), Firmicutes (34%), Proteobacteria (4%), and Verrucomicrobia (4%). Glycopeptide antibiotics, diaminopyrimidine antibiotics, phenicol antibiotics, a fluoroquinolone antibiotic, a tetracycline antibiotic, penam, cephalosporin, and cephamycin were more abundant in the RC group, with about 48% of AROs highly enriched in Proteobacteria (38%) and Firmicutes (10%). Fluoroquinolone antibiotics, disinfectants, and antiseptics, diaminopyrimidine antibiotics, a tetracycline antibiotic, carbapenem, phenicol antibiotics, and macrolide antibiotics were more abundant in the ET group, and about 78% of the AROs were highly enriched in Bacteroidetes (29%), Fusobacteria (23%), Proteobacteria (20%), and Firmicutes (6%). ARGs associated with fluoroquinolone antibiotics, disinfectants and antiseptics, diaminopyrimidine antibiotics, a tetracycline antibiotic, carbapenem, phenicol antibiotics, macrolide antibiotics, penam, cephalosporin, cephamycin, penem, cephalosporin, and monobactam were more abundant in the PJ group. About 78% of the AROs were highly enriched in Bacteroidetes (58%), Proteobacteria (22%), Firmicutes (7%), and Fusobacteria (6%) (Figure 10B–F).

## 4 Discussion

The dietary preferences and nutritional habits of a host species play a crucial role in shaping the composition of its gastrointestinal microbiome (Delsuc et al. 2014; Roggenbuck et al. 2014; Teullet et al. 2023; Xia et al. 2022; Xiao et al. 2022). For example, scavengers derive significant survival advantages from their gut microbiota, which facilitates the detoxification of harmful substances present in decaying organic matter (Roggenbuck et al. 2014). In contrast, insectivores possess a gut microbiota that synthesizes bacterial chitinase, thereby enhancing protein availability (Roggenbuck et al. 2014; Teullet et al. 2023). Similarly, bamboo consumers rely heavily on their gut microbiota to effectively neutralize bamboo cyanide (Xia et al. 2022; Xiao et al. 2022). A study in mice found that a diet too low in carbohydrates resulted in a loss of microbial species diversity (Li et al. 2016).

Based on species‐level LEfSe analysis of gut flora, we found that *Lizard adenovirus A*, *Ovine atadenovirus D*, and *Snake atadenovirus A* were significantly enriched in the RC group. The discovery of these three adenoviruses in the gastrointestinal microbiota of snakes provides further evidence supporting the theory that adenoviruses originated from reptiles (Farkas et al. 2002; Wellehan et al. 2004). *Lizard adenovirus A* has been detected in various lizard species, including *Pogona* spp., *Heloderma suspectum*, and *Heloderma horridum* (Hyndman et al. 2019; Pénzes et al. 2014). The scientific literature lacks research on the impact of *Ovine atadenovirus D* on reptiles (Tang et al. 2012), while *Snake atadenovirus A* has only been observed in studies involving *Elaphe guttata* (Farkas et al. 2008; Singh et al. 2014). Although the specific pathological significance of these three adenoviruses in RC is not fully understood, their discovery enriches our understanding of the diversity of enteroviruses in snakes and lays an important foundation for our next in‐depth systematic study of the snake enterovirus group. In contrast to these adenoviruses, the presence of opportunistic pathogens such as *Plesiomonas*, *Aeromonas*, and *Salmonella* has been observed to be significantly higher in the RC, ET, and PJ, respectively. These pathogens are recognized for their potential to cause intestinal infections or diarrheal diseases in amphibians, reptiles, and humans (Bhunia 2018; Coburn et al. 2007; Kim et al. 2015; Kwon et al. 2019; Marin et al. 2020; Santos et al. 2015). Therefore, a comprehensive assessment of the composition and dynamics of the gut microbial community, including bacteria and viruses, is beneficial to fully understand and monitor the state of gut health in animals.

In the diversity analysis of the gut microbiota in this study, PC exhibited the highest diversity, which was significantly higher than the other three species, followed in order by RC, ET, and PJ. ET and PJ were fed on rodents and birds, so it is assumed that they received prey with a higher protein and fat content, resulting in a lower diversity of the gut microbiota. PC feeds on snails and slugs, and it is assumed that its prey may be enriched in plant fiber, resulting in a higher diversity of the host gut microbiota. The host species under investigation belong to three distinct snake families in the current study. In terms of their phylogenetic relationship, Viperidae and Pareidae are closely related as a sister group, whereas Colubridae is positioned outside of this sister group (Pyron et al. 2013). The Venn diagram analysis revealed that ET and PJ had the lowest number of unique genes but shared the highest number of overlapping genes. The clustering tree and NMDS diagram, based on Bray‐Curtis distance, demonstrated close clustering of samples within the same group. The distance between PJ and ET groups was small, and their clusters were tightly grouped. This finding indicates distinct differences in gut microbiota among groups with varying dietary characteristics. However, ET and PJ, despite being genetically more distant, displayed greater similarity in their gut flora. This suggests that diet may play a significant role in shaping the composition of the gut microbiota.

The gut microbiota observed in the four snake groups at the phylum level aligns with previous findings in *Python bivittatus* , *Agkistrodon piscivorus* , and others (Colston et al. 2015; Kim et al. 2015; McLaughlin et al. 2015; Qin et al. 2019; Shang et al. 2023; Tang et al. 2019; Wei et al. 2023; Zhang et al. 2019). Although stringent decontamination procedures (including surface disinfection, sterile instruments, and a clean bench) were implemented during the experimental process and the predominant microbial taxa align with current research on snake gut microbiomes, a limitation of this study is the absence of negative controls (e.g., sterile water) during sample processing. While it is unlikely that the predominant microbial groups and the beta diversity clustering by group are driven by contamination, this limitation could potentially impact the interpretation of low abundance taxa. Future studies will incorporate negative controls to enhance our understanding of the snake gut microbiome. The dominant bacterial groups observed were Bacteroidetes, Firmicutes, and Proteobacteria, indicating their significant involvement in snake metabolism. Studies indicate that enzymes related to energy metabolism produced by these bacterial groups can effectively degrade a wide range of macromolecules in food, providing a rich source of nutrition for the host (Colston and Jackson 2016; Wu et al. 2011). The Firmicutes demonstrate a wider range of enzymes that aid in the decomposition of carbohydrates and proteins (Zhao et al. 2018). Therefore, a higher Firmicutes/Bacteroidetes (F/B) ratio enables enhanced nutrient absorption from food (Colston and Jackson 2016; Fernandes et al. 2014; Magne et al. 2020; Wu et al. 2011). In this study, we observed that ET and PJ shared similar diets and exhibited comparable F/B ratios. Previous research has also highlighted a clear relationship between the F/B ratio and the dietary environment in animals (Murphy et al. 2010; Zhong et al. 2022). Additionally, the abundance of Firmicutes was found to be higher in the PC group compared to the other groups. Notably, the PC group exhibited a high abundance of Verrucomicrobia. This bacterial group, commonly associated with soil and freshwater environments (Lee et al. 2009). When ingesting snails/slugs, the PC group may ingest environmental microorganisms attached to their body surface, which in turn affects their gut flora. The PC group may ingest environmental microorganisms attached to the body surface of snails/slugs while feeding on them, thus affecting their gut microbiota composition.

The level of Bacteroidetes was found to be significantly higher in the PJ group compared to the ET group. Bacteroidetes are known for their ability to break down polysaccharides, enhance nutrient absorption, improve digestion, and increase the utilization of complex carbohydrates. Additionally, they play a role in lactose fermentation, and various metabolic processes, and contribute to the formation of intestinal mucosa (Stappenbeck et al. 2002). LEfSe analyses were also carried out that found that PJ was the most enriched in carbohydrate metabolism pathways. Notably, Fusobacteria were higher in the ET and PJ groups. This phylum is linked to protein catabolism in scavengers consuming high‐nutrient resources (Colston and Jackson 2016). The large prey size of ET and RJ, combined with the prolonged feeding intervals typical of snakes, suggests that the Fusobacteria augment the degradation efficiency of prey proteins, thereby enabling efficient nutrient extraction. Previous studies in *Alligator mississippiensis* , *Coragyps atratus* , *Cathartes aura* , *Ictalurus punctatus* , *Micropterus salmoides* , and *Lepomis macrochirus* suggest that Fusobacteria are involved in amino acid metabolism or affect luminal biofilm development (Keenan et al. 2013; Larsen et al. 2014; Mira et al. 2004). There are valid reasons to support the notion that the gut microbiota of snakes significantly contributes to digestion and absorption processes and that the composition of the gut microbiota is heavily influenced by dietary patterns.

The composition of the gut microbiota has a strong correlation with food characteristics (Brunetti et al. 2023). Diets that are abundant in carbohydrates have been observed to exhibit increased levels of *Prevotella*, whereas diets rich in protein and fat tend to be predominantly dominated by *Bacteroides* (Khan et al. 2018). In this study, the top 10 most abundant genera across four snake species included Bacteroides, which aligns with their dietary preference for high‐fat and protein‐rich foods as carnivores. Additionally, both Bacteroides and Salmonella were dominant genera in the ET and PJ groups. We hypothesize that this shared dominance is related to their similar dietary habits. However, we observed notable variations in the primary gut microbiomes among the groups. Specifically, the PC group displayed a higher prevalence of *Alistipes*, a genus associated with thriving on high–fat diets and avoiding ones rich in plant foods, as indicated by related studies (Khan et al. 2018). The MetaStats analysis revealed that the PC group exhibited significantly increased activity in liposome metabolism and cholesterol metabolism pathways compared to the other three groups. This finding suggests the possibility of an exclusive microbiota lineage, represented by *Alistipes*, associated with slug– and snail–eating individuals. However, further investigation is necessary to gain a deeper understanding of this relationship. *Parabacteroides*, recognized for their extensive capacity in carbohydrate utilization (Cui et al. 2022), contributes to this important metabolic process. Additionally, the gut of the RC group exhibited a high enrichment of *Clostridium*, which previous research has shown is influenced by the consumption of dietary carbohydrates and proteins (Guo et al. 2020); meanwhile, we observed variances in the amino acid substitution pathways during our LEfSe analyses. Proteobacteria represented the dominant phylum in the gut microbiota of the RC group, including *Providencia* within its classification. Previous research in *Caenorhabditis elegans* has demonstrated the ability of Providencia to produce biologically active neurotransmitters. These neurotransmitters have been found to influence the sensory behavior of the host organism and impact its feeding patterns (O'Donnell et al. 2020). *Cetobacterium* is highly enriched in the gut of the ET group, recognized as a core microbiota in carnivorous fish (Cui et al. 2022; Nayak 2010). Its abundance showed a negative correlation with *Bacteroides*, indicating a potential diet–driven balance regulation (Hao et al. 2017). Interestingly, during our observations, we noted that Bacteroides were found to be more prevalent in PJ with similar dietary properties. It is worth mentioning that an increase in *Bacteroides* has been linked to obesity in animals, possibly due to their capacity to extract additional energy from food that is typically difficult to digest (Turnbaugh et al. 2006). These findings suggest that the bacterial composition observed in this snake suggests that it may possess an enhanced ability to efficiently extract additional nutrients from its prey.

The composition of the gut microbiota is significantly influenced by diet (Xia et al. 2022; Xiao et al. 2022; Zheng et al. 2022). Given that all snakes adhere to a carnivorous diet characterized by high protein and low carbohydrates (Holmberg et al. 2002), our primary focus lies in studying the amino acid and carbohydrate metabolism pathways that are essential for the digestion and absorption of food (Zheng et al. 2022). Meanwhile, the four snake species also showed a high abundance of carbohydrate transport and metabolism, amino acid transport, and metabolism. LEfSe analysis at the KEGG level 3 shows significant differences in a total of 28 pathways, with the differences mainly concentrated in the amino acid and carbohydrate metabolism pathways. Of these, the amino acid and carbohydrate metabolism pathways of PC are the most abundant, and presumably, the PC diet is the most unique. For example, GH18 represents a chitinase, and chitin is abundant in molluscan organs and epidermis (Muzzarelli et al. 2012), suggesting a key role of diet in shaping the snake gut microbiota, and a variety of enzymes that promote the breakdown of carbohydrate macromolecules into small molecules were found to be more abundant in PC than in other groups of snakes. Specific enzymes such as GH19 (for the enzyme chitinase) were also detected in RC in this study, and it is hypothesized that the presence of this enzyme helps the host digest chitin in the epidermis of firefly larvae (O'Donnell et al. 2020). The above results indicate that there are notable differences in the prevalence of genes related to amino acid and carbohydrate metabolism pathways associated with dietary preferences in the digestive systems of snakes. However, our study did not find evidence of increased gene specificity in ET for pathways associated with amino acid and carbohydrate metabolism, as compared to PJ. Although PJ is a venomous snake, venom aids in the digestion of prey (Blaylock 2002; Van der Walt and Joubert 1971). Studies on *Bitis arietans* , *Oxyuranus scutellatus* , and *Crotalus atrox* have shown that venom hydrolyzes casein and other proteins, increasing digestibility, even in live mice (Blaylock 2002; Nicholson et al. 2006; Thomas and Pough 1979; Van der Walt and Joubert 1971). Due to the narrower feeding range of ET compared to PJ, we initially expected that ET would show higher gene specificity in amino acid and carbohydrate metabolism pathways compared to PJ. Surprisingly, the results of our study completely contradicted this hypothesis. Therefore, it is suggested to further investigate the metabolic role of the gut microbiomes in both PJ and ET, possibly using additional methods.

The emergence of antibiotic‐resistance genes (ARGs) is an important public health problem worldwide, and intestinal flora is the main source of ARGs (Nicholson et al. 2006; Zeng et al. 2019). Currently, the use of antibiotics is more common in clinical, poultry, and livestock disease prevention and treatment, and the frequent use of antibiotics leads to antibiotic‐selective pressure on the organism and contributes to the spread of antibiotic resistance (Schechner et al. 2013). There is a strong association between the food chain and ARGs (Kumar et al. 2020). For example, the EU's ban on avoparcin in animal feed has reduced the prevalence of vancomycin‐resistant enterococci (VRE) in both the general population and animals (Hawkey 2008). In our study, we found that RC demonstrated a higher prevalence of genes associated with resistance to antibiotics in metabolic pathways compared to other species. Our sampling records showed that RC in this study were collected from maize fields or grasslands close to villagers' houses, which may be related to the widespread use of antibiotics in poultry, livestock, and agriculture. Further analysis indicated a correlation between the spectrum of ARGs and the composition of the microbiota community. Specifically, the predominant phyla Firmicutes and Proteobacteria were identified as the main sources of ARGs in RC. Interestingly, our research revealed that there may be a correlation between antibiotic‐resistant genes and the food chain. We observed that earthworm and snail/slug–feeding reptiles had a higher abundance of antibiotic‐resistant genes compared to rat–feeding reptiles and other species. Soil invertebrates such as earthworms and snails are exposed to contaminated organic matter due to their feeding behavior, and their intestinal tracts become enrichment sites for environmental ARGs (Ding et al. 2019; Zhang et al. 2025). In contrast, ET and PJ prey are less exposed to environmental ARGs, and the vertebrate gut environment may attenuate the transmission of exogenous ARGs (Kumar et al. 2020). Consequently, the resistant flora and the ARGs they carry are passed on from prey to predators. Dietary habits further influence the maintenance of ARGs by shaping the structure of the gut flora and selective pressure.

## 5 Conclusions

This study constitutes the inaugural extensive examination of the gut microbiota across four snake species with diverse dietary preferences, employing metagenomic approaches. The findings can be summarized as follows: (1) The core structure of the snake gut microbiota is closely associated with dietary preferences, which effectively facilitates the digestion of carbohydrates and proteins in their diet. Functional enrichment analyses revealed significant differences in carbohydrate and amino acid metabolic pathways among the four snake species. Notably, *Pareas chinensis* exhibited a higher prevalence of genes encoding carbohydrate‐activated enzymes, suggesting that this species demonstrates a more distinct dietary preference compared to the other three groups. (2) Two adenoviruses, identified as Lizard *atadenovirus A* and *Ovine atadenovirus D*, were detected for the first time within the intestinal microorganisms of *Rhabdophis chiwen*. (3) Antibiotic resistance genes are commonly found in the gut microbiomes of all four snake species studied, including those conferring resistance to tetracycline and fluoroquinolone antibiotics. The results suggest the efficacy of disparate diets in resolving potentially pertinent host characteristics, thereby offering insights into the associations by which gut microbes assort among host species.

## Author Contributions

**Huina Song:** conceptualization (equal), data curation (equal), writing – original draft (supporting), writing – review and editing (equal). **Xiufeng Li:** conceptualization (equal), data curation (equal), writing – original draft (equal), writing – review and editing (equal). **Jingxue Luo:** investigation (equal), writing – review and editing (equal). **Ji Wang:** investigation (equal), writing – review and editing (equal). **Fei Wu:** methodology (equal), writing – review and editing (equal). **Jiuyan Jiang:** methodology (equal), writing – review and editing (equal). **Ji Chen:** methodology (equal), writing – review and editing (equal). **Yuqi Cheng:** methodology (equal), writing – review and editing (equal). **Yujia Yong:** methodology (equal), writing – review and editing (equal). **Mingwen Duan:** methodology (equal), writing – review and editing (equal). **Guangxiang Zhu:** data curation (equal), funding acquisition (equal), project administration (equal), resources (equal), supervision (equal), writing – original draft (equal), writing – review and editing (equal).

## Ethics Statement

Snakes were collected and sacrificed by IACUC protocols approved by the Institutional Animal Care and Use Committee of the Sichuan Agricultural University under permit number Approval No. 20210071. The euthanasia of the animals took their welfare into full account. This study did not involve any endangered animals.

## Conflicts of Interest

The authors declare no conflicts of interest.

## Supporting information


**Table S1:** Raw and Clean reads from the analyzed samples.

**Table S2:** Assembly results for each sample.

**Table S3:** ORF prediction results.

**Table S4:** Relative abundance of classified gut microbiome in samples.

**Table S5:** Classification of gut microbiome at the genus level.

**Table S6:** Relative abundance of ARO of top10 in each sample.

## Data Availability

The data supporting the findings of this study are publicly available from the National Center for Biotechnology Information (NCBI) Sequence Read Archive (SRA) at https://www.ncbi.nlm.nih.gov/bioproject/PRJNA1088789; the reference number is PRJNA1088789.
